# Supplementary material for: The Impact of the COVID Pandemic on the Incidence of Presentations with Cancer-Related Symptoms in Primary Care
Source: Cancers (Basel). 2022 Oct 30;14(21):5353. doi: 10.3390/cancers14215353 (PMC9656532; doi:10.3390/cancers14215353)
Supplement: Supplementary file 1 [file cancers-14-05353-s001.zip › cancers-1914235-supplementary.pdf]

Supplementary files

**Cancer-related symptoms**	**History of cancer**	**Cardiovascular Disease**	**Psychiatric / Psychological**
- A04 – tiredness	- S77.03 – melanoma	- K71 – rheumatic heart disease	- P01 - anxiety
- B02 – lymphadenopathy	- X76 – breast cancer	- K74 – angina pectoris	- P02 – acute stress reaction
- D13 – jaundice	- D75 – colorectal cancer	- K75 – myocardial infarction	- P03 – depressed
- D15 – melaena	- R84 – lung cancer	- K76 – ischaemic heart disease	- P15 – chronic alcohol misuse
- D16 – rectal bleeding	- A79 – cancer unknown primary	- K77 – heart failure	- P19 – drug misuse
- D18 – change in bowel habits	- B74 – haematological cancer	- K83 – cardiac valve disease	- P29 – other psychiatric symptoms
- D21 – swallowing problems	- D74 – stomach cancer	- K84 – other heart disease	- P70 – dementia
- D24 – abdominal mass	- D76 – pancreatic cancer	- K85/86/87 – hypertension	- P71 – organic psychosis
- S82 – naevus	- D77 – Upper-gastrointestinal cancer	- K89 – transient cerebral ischaemic	- P72 – schizophrenia
- T08 – weight loss	- L71 – nervous system cancer	- K90 – stroke	- P73 – affective psychosis
- U06 – haematuria	- R85 – respiratory cancer	- K91 – atherosclerosis	- P74 – anxiety disorder
- X12 – postmenopausal bleeding	- T71/73 – endocrine cancer	- K92.01 – claudication	- P76 – depression
- X19 – breast lump	- U75/76/77 – urinary cancer	- K93 – lung emboli	- P77 – suicide attempt
	- Y77 – prostate cancer	- K94 – thrombophlebitis	- P78 - neurasthenia
	- Y78 – male genital cancer	- K99 – cardiovascular disease other	- P79 – phobia / compulsion
		- W77.03 – deep vein thrombosis	- P80 – personality disorder
**Diabetes**	**Chronic Obstructive Airways Disease (COPD)**		- P85 – mental handicap
T90 – diabetes mellitus	- R95 - COPD		- P98 – other psychosis

Supplementary Table S1: ICPC codes

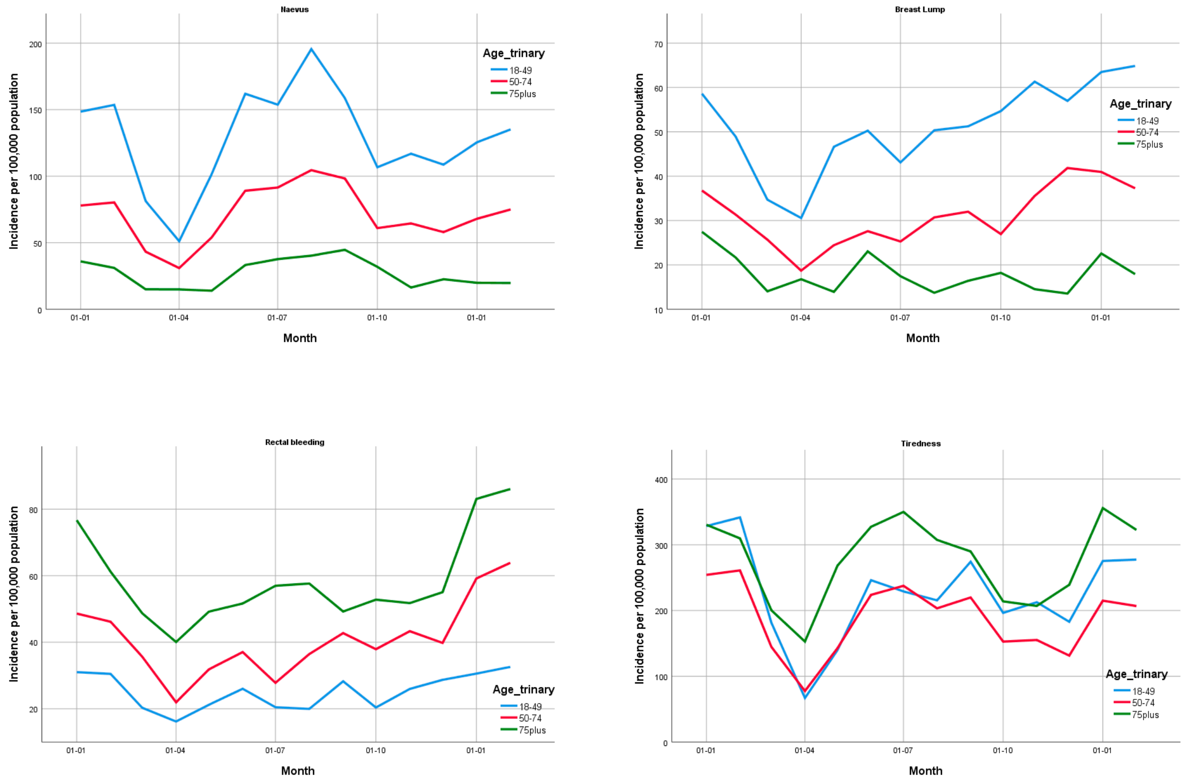

Supplementary Figure S1: Incidence according to age

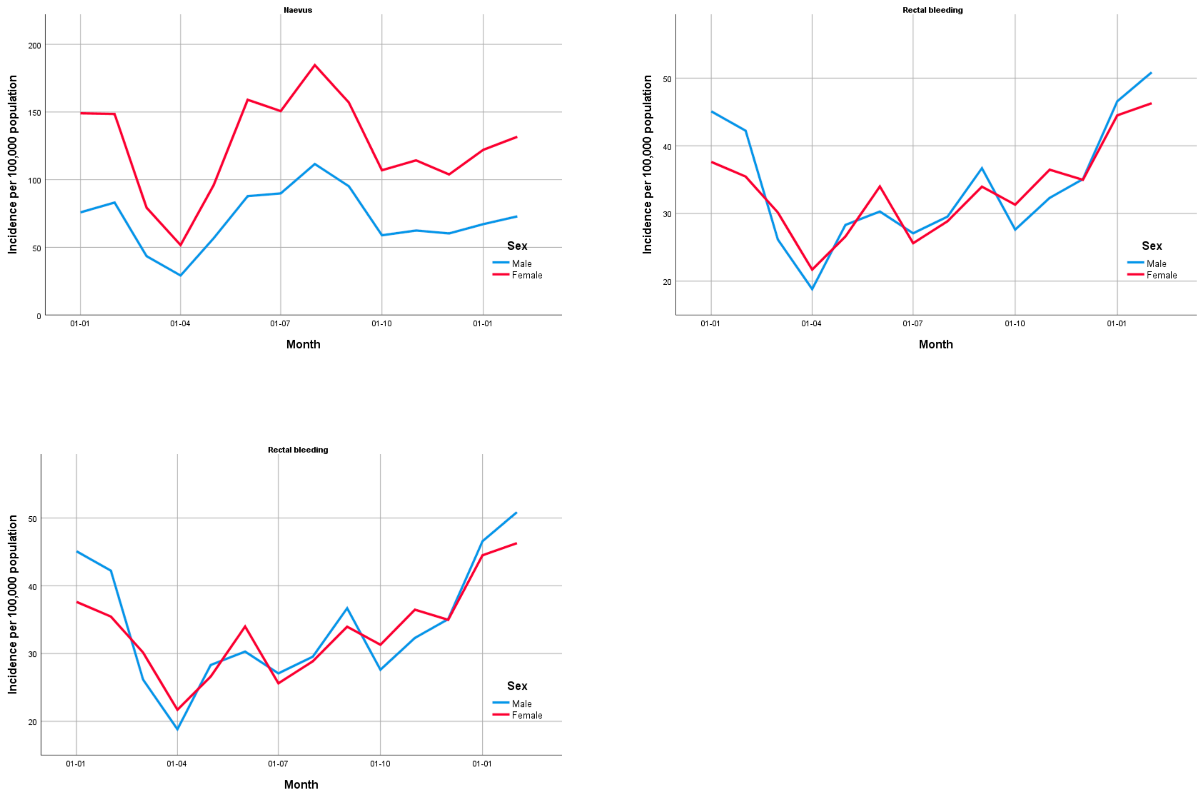

Supplementary Figure S2: Incidence according to sex

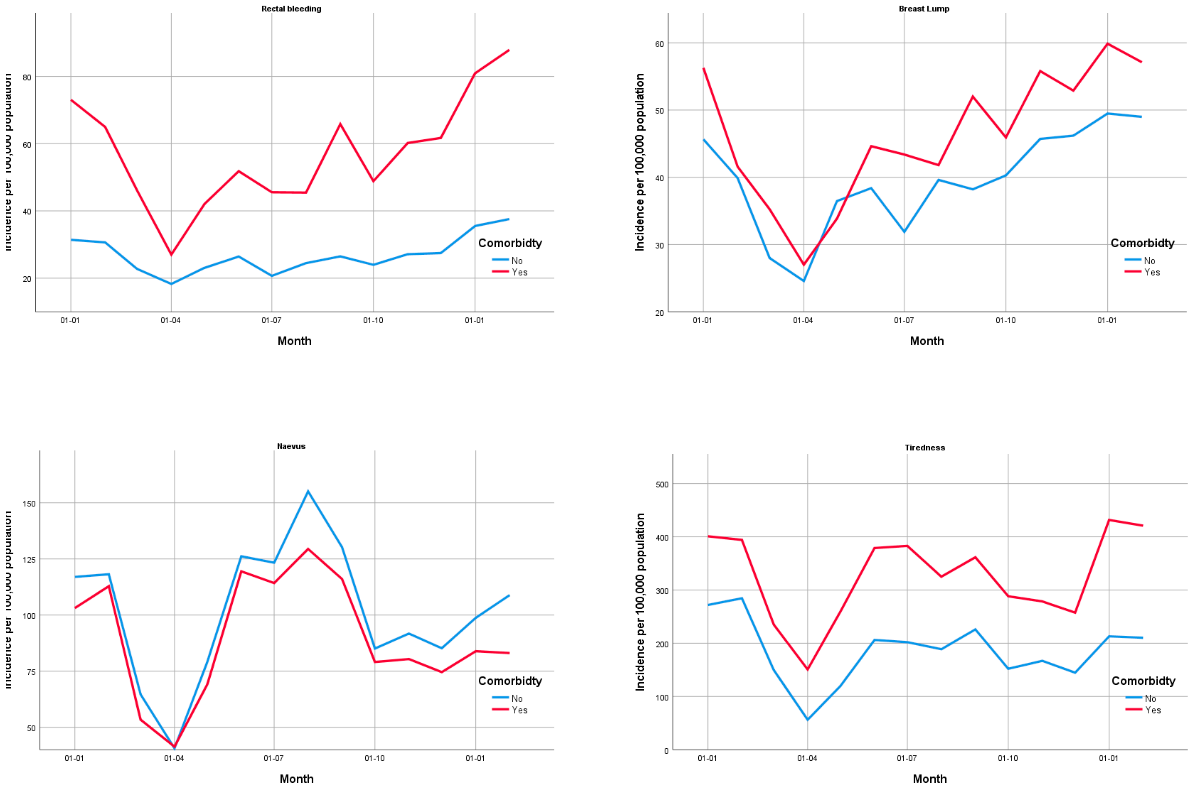

Supplementary Figure S3: Incidence according to major comorbidity status (defined as cardiovascular, diabetes, or chronic obstructive airways disease, and psychiatric or psychological diagnosis at any timepoint, coded through ICPC codes described in supplementary file, Table S1.

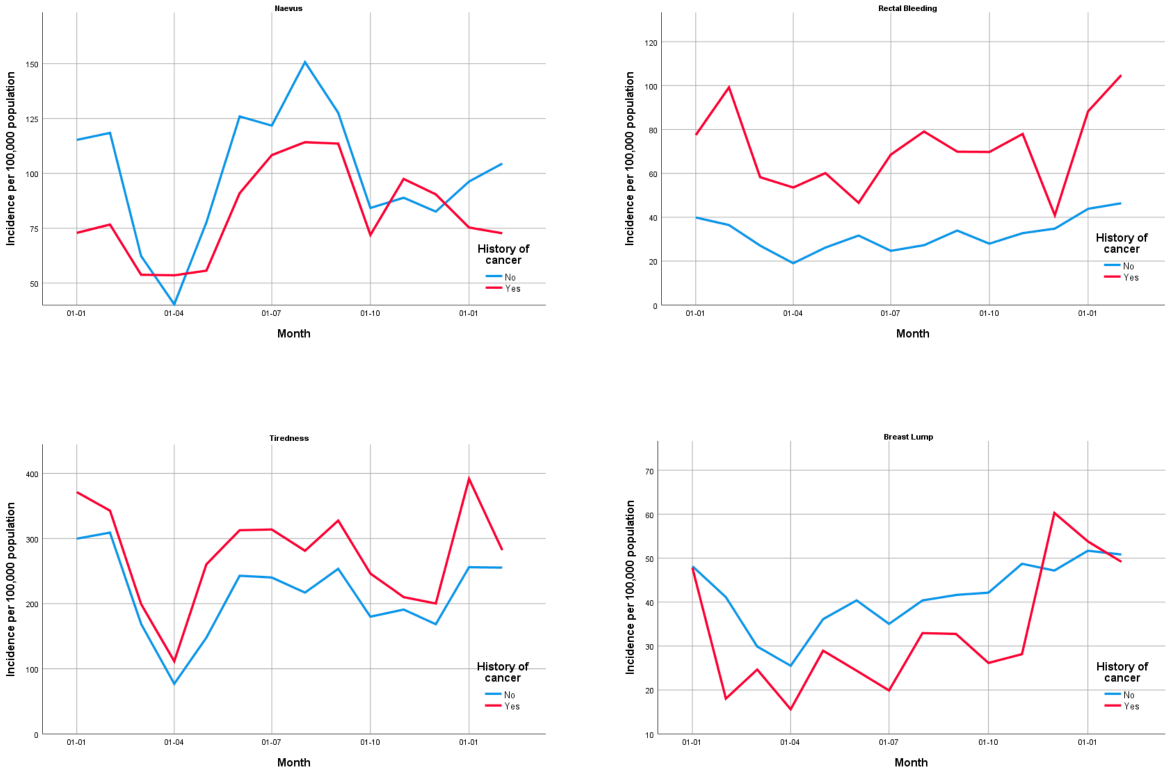

Supplementary Figure S4: Incidence according to history of cancer
